# ANN-Based Instantaneous Simulation of Particle Trajectories in Microfluidics

**DOI:** 10.3390/mi13122100

**Published:** 2022-11-28

**Authors:** Naiyin Zhang, Kaicong Liang, Zhenya Liu, Taotao Sun, Junchao Wang

**Affiliations:** 1 School of Automation, Hangzhou Dianzi University, Hangzhou 310018, China; 2 Key Laboratory of RF Circuits and Systems, Ministry of Education, Hangzhou Dianzi University, Hangzhou 310018, China

**Keywords:** microfluidics, machine learning, particle trajectory, design automation, computer-aided design

## Abstract

Microfluidics has shown great potential in cell analysis, where the flowing path in the microfluidic device is important for the final study results. However, the design process is time-consuming and labor-intensive. Therefore, we proposed an ANN method with three dense layers to analyze particle trajectories at the critical intersections and then put them together with the particle trajectories in straight channels. The results showed that the ANN prediction results are highly consistent with COMSOL simulation results, indicating the applicability of the proposed ANN method. In addition, this method not only shortened the simulation time but also lowered the computational expense, providing a useful tool for researchers who want to receive instant simulation results of particle trajectories.

## 1. Introduction

As an emerging technology in biological analysis, microfluidics has shown great potential in isolating cells and enriching rare cells in the past decades due to the advantages of the small quantity of sample consumption, ease of operation, and high accuracy [1]. Researchers have developed different types of microfluidic cell sorters for separating cells such as circulating tumor cells [2,3], platelets [4,5,6], T-cells [7,8], target single cells [9,10], and other biological markers from blood samples or cell suspensions extracted from interest biological areas [8,11,12]. The sorting methods are developed based on different properties of the cells such as size [13,14] and affinity [7,8], or different technologies such as dielectrophoresis [5], magnetic activation [15], fluorescence activation [16,17], image recognition [18,19], vortex [20], and acoustic waves [3,6,16].

The above-mentioned microfluidic cell sorters provide sufficient solutions to the target goals, however, it is undeniable that those devices are usually designed by hand based on the experience of researchers, which can take months or even years to design. For example, in a deterministic lateral displacement (DLD) sorter, the target cells can differ in size, morphology, and affinity to fluorescence dyes as well as fluorescence intensities, which makes it extremely important to design proper pillar arrays for cell detection and separation [21]. These design units include pillar shape, size, angle, the distance between each other, the length of the path, etc., and the whole process can be repeated multiple times from design, fabrication, test, and design revision to correct cell flow trajectory, which is time-consuming and labor-intensive. Therefore, an economic method to predict cell trajectories becomes an urgent need. Researchers have developed tools utilizing computer simulation such as finite element analysis (FEA) to test and optimize their designs before chip fabrication [22,23,24]. In simulations, the biological cells can be treated as particles with parameters mimicking reality, while the particle trajectory simulation result can be used to optimize the design in return until a qualified chip structure is obtained.

However, the FEA method for simulating particle behaviors in microfluidic devices is not only computationally expensive and labor-intensive but also hard for researchers to learn and use. Specifically, you need to use the Laminar Flow module to solve the velocity field, then use the Particle Tracing module to solve the cell trajectory, which has a steep learning curve for engineers, and takes a long time for researchers to fully master without considering lots of tricky configurations in COMSOL. A promising solution is applying machine learning techniques to accelerate the simulation process by replacing the FEA simulation kernel [25,26,27]. Among all machine learning techniques, artificial neural networks (ANNs) play an important role in highly complex particle trajectory analyses [28,29]. Briefly, an ANN is based on a collection of connected units or nodes called artificial neurons, which loosely model the neurons in a biological brain. The ANN receives signals (input data) and then processes (does a few calculation steps) to generate output signals as output data. The output data will have different physical meanings in the corresponding fields such as computer vision [30], natural language process [31], and pattern recognition [32].

To partially address the above issue, a simulation method was presented in our previous work that decomposed microfluidic devices into units including channels and intersections and constructed a database containing almost ten thousand pre-simulated channel intersections [33]. That work has accelerated the simulation process and lowered the need for advanced computational devices, but the limitations of FEA still exist. Therefore, a cloud database was constructed subsequently using an improved method inspired by dynamic programming to accelerate simulations of particle behaviors in the channels [34]. In this work, a further step was taken by increasing the computational rate and reducing the computational time by leveraging machine learning techniques. Specifically, an ANN was proposed to predict the instant particle trajectory at critical intersections in microfluidic devices.

## 2. Theory of the Design

**Figure 1:**
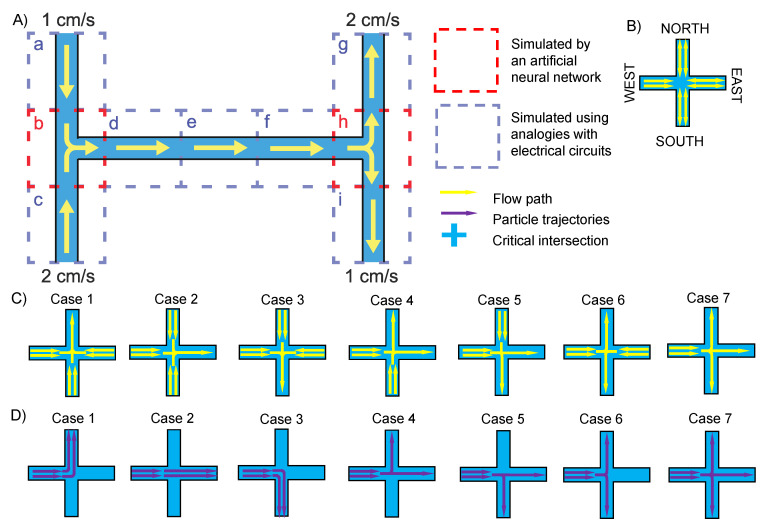
Theory of the design. (**A**) A basic “H” channel chip is decomposed into nine units (a to i) to demonstrate the straight channels and critical intersections. Units a, c, d, e, f, g, and i are straight channels, while units b and h are critical channel intersections. (**B**) Schematic of critical intersections. (**C**) Seven cases illustrating the fluid behavior at critical intersections. (**D**) Seven cases illustrating the particle behavior at critical intersections.

In most microfluidic devices, the channels are classical “H” channels and can be decomposed into two types: straight channels and intersections (Figure 1A). In those straight channels, particles usually flow along the channel with their trajectories showing straight lines (Figure 1A (unit a, c, d, e, f, g, and i)), which is akin to the flow of electricity in a network of resistors and can be modeled using principles of electrical circuit analysis. Therefore, particle behaviors at critical intersections are crucial to be analyzed and their trajectories to be predicted (Figure 1A (unit b, h)). As a result, the complete particle trajectory can be computed by piecing the trajectories in the channels and at the intersections. In our previous work, the FEA method was used to predict the particle behavior at critical intersections but not the whole chip [33]. In this work, an ANN method was proposed to predict particle trajectory at critical intersections as shown in the red squares in Figure 1A (unit b, h), and the complete particle trajectory was computed by using the piecing method. The whole process using the ANN can greatly reduce computational time and have low or even zero cost in computation. The schematic of the critical intersection is defined in Figure 1B, which has four ports named the East, West, South, and North ports. For the West port, the fluid can only flow in, while for the other three ports, the fluid can both flow in and out. The fluid behavior at critical intersections can be illustrated in seven cases as shown in Figure 1C. For example, in case 1, the fluid can flow into the channels from the East port, West port, and South port, while flowing out from the North port. There are also seven cases to illustrate the particle behavior at critical intersections as shown in Figure 1D; however, the particle behavior is not consistent with the fluid behavior. For example, the particle behavior in case 1 can happen in the flow behaviors in cases 1, 4, 6, and 7. Consequently, it is important to analyze both the fluid behavior and particle behavior specifically.

## 3. Materials and Methods

**Figure 2:**
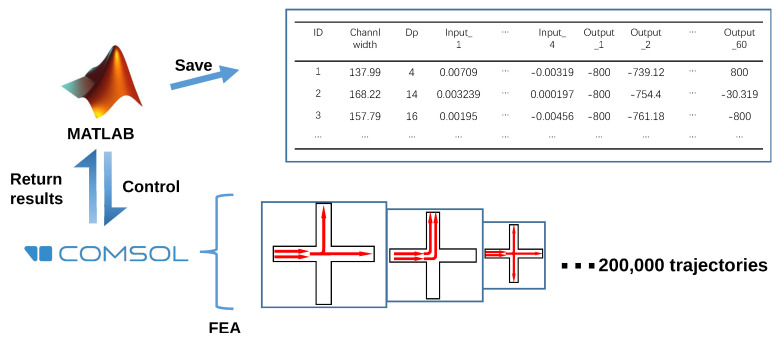
Data generation flow chart. MATLAB was used to automate the COMSOL simulation process.

Supervised learning was applied to train the proposed ANN. In order to construct the training set as the very first step, MATLAB was used to control COMSOL to generate the database. As shown in Figure 2, the West port of the critical intersection shown in Figure 1 was set to be an inlet only, while the other three ports could be configured as both inlets or outlets in this simulation process. To generate random cases of the critical intersection, the inflow rates of the fluid were randomly varied from 0 to 2 cm/s, and for an intersection with outlet number of N = 1, 2, or 3, the outflow rates of N − 1 of the outlets were randomly varied from 0 to 2 cm/s. To guarantee mass conservation, the outflow rate of the remaining outlet was defined to be the total inflow rate minus the total outflow rate. Particle diameter was a variable ranging from 1 to 20 μm, set to be the input, and channel width was a variable ranging from 50 μm to 200 μm. Using this approach, 200,000 different particle trajectories at critical intersections were predicted.

Simulation of the 200,000 microfluidic intersections was conducted in COMSOL Multiphysics and the COMSOL LiveLink API for MATLAB was used to automate the process. The Laminar Flow physics module with a customized mesh of 1 μm maximum mesh size was used to solve the fluid velocity field at each intersection. The simulation results were confirmed to be not altered by finer meshes, which demonstrated mesh independence. The inlet and outlet boundary conditions were defined as introduced in the above paragraph. The other boundaries were defined as walls with no-slip boundary conditions, and the channels were filled with water under incompressible flow. A stationary solver was used to calculate the velocity field.

The path of particle flow through each intersection in our database was calculated using the Particle Tracing for Fluid Flow physics module in COMSOL Multiphysics. A “Drag Force” boundary condition was added to each channel, a particle “Inlet” boundary condition with “Uniform distribution” of initial positions was added to all inlets (10 particles per release), and the rest of the channels were assigned “Outlet” boundary condition. The walls were assigned the “Freeze” boundary condition, meaning that particles in contact with the channel walls would stick there, which is a realistic assumption in microfluidic devices. The simulation process was repeated for each intersection using particles with diameters ranging from 1 μm to 20 μm. The resulting 200,000 simulated particle trajectories were stored in the simulation database. A time-dependent solver was used for the calculation of the particle trajectory.

**Figure 3:**
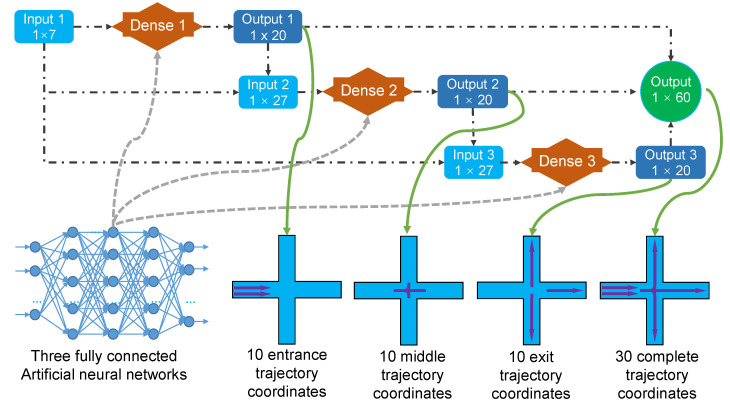
The structure of the proposed ANN.

The proposed ANN is physics-informed and the schematic of ANN training is shown in Figure 3. Three dense layers were used to train the target ANN and each dense layer was a fully connected ANN, predicting part of the particle trajectory. Specifically, the first dense Input 1 had 1 × 7 parameters, which were particle diameters (Dp), channel width, the initial coordinates x0 and y0 of the particle, and the inflow rate or outflow rate of the West, North, and East ports. Output 1 was the coordinates of the first ten points of the simulated entrance trajectory (1 × 20). Input 2 for dense 2 was composed of the 7 parameters of Input 1 and 20 outputs of Output 1, which was 27 parameters (1 × 27). There were also 20 outputs at Output 2, which were the coordinates of the first ten points of the simulated middle trajectory (1 × 20). Input 3 for dense 3 was composed of the 7 parameters of Input 1 and 20 outputs of Output 2, which was 27 parameters (1 × 27) as well. Similarly, there were also 20 outputs at Output 3, which were the coordinates of the first ten points of the simulated exit trajectory (1 × 20). The 1 × 20 parameters at Outputs 1, 2, and 3 provided the total 1 × 60 outputs. The three dense layers were three fully connected ANNs, and the complete particle trajectory was obtained by piecing the entrance, middle, and exit trajectories. A more detailed configuration of the dense network in the proposed ANN can be found in Table 1.

The training process was implemented in Python 3.10 with the help of PyTorch. The loss during the training process was calculated using mean squared error (MSE), and the accuracy rate was determined by using R square.

## 4. Results and Discussion

**Figure 4:**
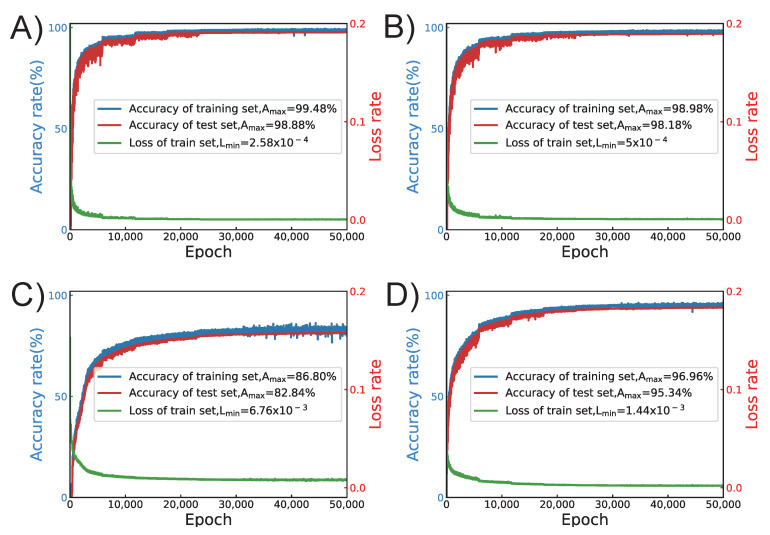
The training curve of the proposed ANN using four different activation functions. (**A**) Leaky ReLU function. (**B**) ReLU function. (**C**) Sigmoid function. (**D**) Tanh function.

The training process and the performance of the proposed ANN using four different activation functions are presented in Figure 4. In total, 70% of the dataset is used as the training set, and the other 30% is used as the test set. As it is shown in Figure 4A, using the Leaky ReLU as the activation function, the loss rate is $2.58\times 10^{-4}$, the accuracy rate of the training set is 99.48%, and the accuracy rate of the test set is 98.88% after training for 50,000 epochs. Figure 4B shows the training result using the ReLU function, the loss rate is $5.00\times 10^{-4}$, the accuracy rate of the training set is 98.98%, and the accuracy rate of the test set is 98.18% after training for 50,000 epochs. Figure 4C shows the training result using the Sigmoid function, the loss rate is $6.76\times 10^{-3}$, the accuracy rate of the training set is 86.80%, and the accuracy rate of the test set is 82.84% after training for 50,000 epochs. Figure 4D shows the training result using the Tanh function, the loss rate is $1.44\times 10^{-3}$, the accuracy rate of the training set is 96.96%, and the accuracy rate of the test set is 95.34% after training for 50,000 epochs. There are obvious differences when comparing the four groups of results. The loss rate, the accuracy rate of the training set, and the accuracy rate of the test set all decreased from the proposed ANN method using the Leaky ReLU as the activation function. The next best was ReLU, then Sigmoid, and then Tanh. In a word, the three parameters all achieved the highest in the ANN method using the Leaky ReLU function. Therefore, the Leaky ReLU function was chosen as the activation function in the proposed ANN.

**Figure 5:**
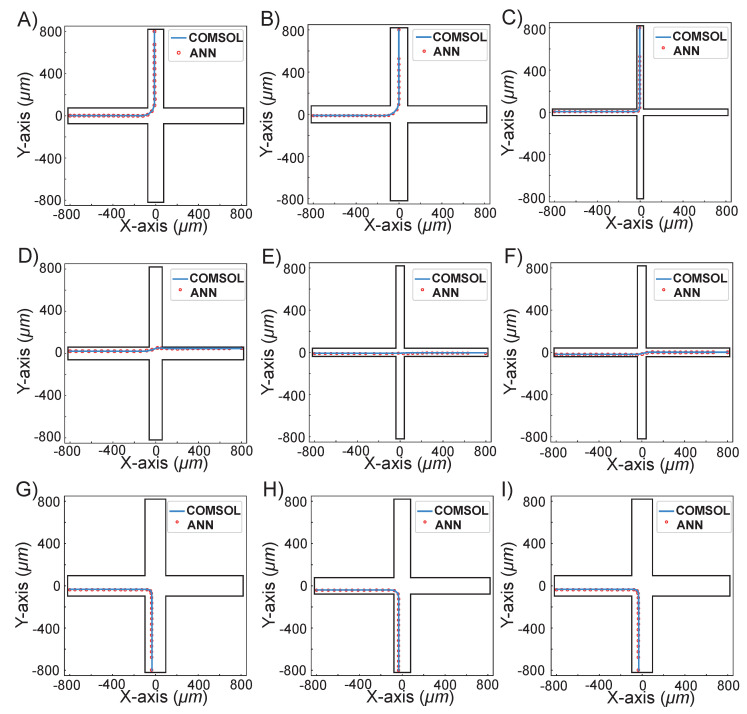
The nine randomly selected simulation results showing the comparison between ANN-predicted and COMSOL-predicted particle trajectories.

Figure 5 demonstrates nine randomly selected cases simulated using our proposed ANN method compared with the COMSOL method. The detailed parameters of nine randomly selected cases are shown in Table 2. It can be seen from both Figure 5 and Table 2 that case A and case G all had particle diameters of 13 μm, and similar channel widths of 190.34 and 194.02 μm, respectively. Although the particle flowing path was input from the West and output from the North for case A, and input from the West and output from the South for case G, the particle trajectories predicted with the proposed ANN method were both highly consistent with the COMSOL method. In case H and case I, the input particle diameter was 11 μm, while the channel widths were 156.21 and 72.04 μm, respectively. In those two cases, the particle flowing path was closer to the wall in case H while farther from the wall in case I, which showed that the input position of the particle does not affect the ANN prediction results. In other cases, D, E, and F had three different channel widths and particle diameters, and as a result, the particle flow paths varied from the upper wall, to the middle channel, and to the lower wall, which showed that the particle diameter and channel width had no effect on the ANN prediction results. Cases B and C were consistent with the above phenomenon. The nine randomly selected cases showed that no matter what the value of particle diameter and the channel width is, the particle trajectories predicted by our ANN method are always highly consistent with COMSOL predictions.

### Potential Limitations

The proposed ANN prediction method showed good results that match the COMSOL simulation method. However, there are still some potential limitations when using this ANN method. First of all, this ANN method is only adapted for rectangular intersections currently, other structures such as circular intersections or angles between channels less or more than 90 degrees are not applicable. Secondly, this method is only applied for low Re number laminar flow cases, while the turbulence flow cannot be predicted with this ANN method. The third limitation is that the resolution of the method is fixed, with only 60 points per trajectory, which might not be enough for some cases requiring more data points. Our future work will be dedicated to solving the above issues.

In addition, the overall simulation time in COMSOL is usually minutes or even hours depending on different models, while the ANN processing time is usually at the millisecond level no matter what kind of the model is. Therefore, it is not so meaningful to compare the specific computational time for these two methods. However, for most microfluidic chip simulation models, the simulation cost is reduced from several hours or minutes to several seconds or even the millisecond level.

## 5. Conclusions

In this work, an ANN method was proposed to facilitate the microfluidic device design process and lower the cost, and the results showed high consistency with the COMSOL prediction method. The ANN method with three dense layers was used to solve particle trajectories at the critical intersections, and in the future, it can be further applied to accelerate the simulation process of particle trajectory in the whole chip, by taking advantage of analogy circuits. With our current method, the simulation cost is reduced from several hours or minutes to several seconds, which is 100× or even 1000× faster. Although there are still some limitations using the current ANN method, we are confident to work on it and solve the issues, bringing a more advanced ANN tool to accelerate the design process for researchers to invent new cell sorting chips for many different applications.

## Figures and Tables

**Table 1:** The configuration of the proposed dense network.

ID	Layer	Dense 1 (Neurons)	Dense 2 (Neurons)	Dense 3 (Neurons)	Activation
1	Input	7	27	27	Linear
2	Fully connected	400	400	400	Leaky ReLU
3	Fully connected	400	400	400	Leaky ReLU
4	Fully connected	400	400	400	Leaky ReLU
5	Fully connected	400	400	400	Leaky ReLU
6	Fully connected	400	400	400	Leaky ReLU
7	Output	20	20	20	Linear

**Table 2:** The detailed parameters of nine randomly selected cases.

ID	Channel Width (μm)	Particle Diameter (μm)
A	190.34	13
B	161.23	11
C	62.36	10
D	120.63	17
E	78.65	10
F	82.36	18
G	194.02	13
H	156.21	11
I	72.94	11

## Data Availability

Not applicable.
